# Shared breath of joy enhances empathy through breathing synchronization

**DOI:** 10.1038/s41598-026-34981-0

**Published:** 2026-01-05

**Authors:** Yuri Masaoka, Motoyasu Honma, Momoka Nakayama, Misako Matsui, Akira Yoshikawa, Shota Kosuge, Miku Kosuge, Daiki Shoji, Shunsuke Sakakura, Masahiko Izumizaki

**Affiliations:** 1 Department of Physiology, Showa Medical University School of Medicine, 1-5-8 Hatanodai, Shinagawa-ku, Tokyo, 142-8555 Japan; 2 Dentsu Lab Tokyo, Tokyo, Japan; 3 Division of Health Science Education, Showa Medical University School of Nursing and Rehabilitation Sciences, Yokohama, Japan; 4 Division of Neurology, Showa Medical University Hospital, Tokyo, Japan; 5 Department of Respiratory Medicine, Showa Medical University Fujigaoka Hospital, Yokohama, Japan

**Keywords:** Breathing, Synchronization, Empathy, Familiarity, Emotions, Joy, Human behaviour, Quality of life

## Abstract

Physiological synchronization has been observed in empathy. However, the specific role of breathing synchronization in emotional states remains unexplored. Here, we found that synchronized breathing that matches the observation of another person’s joyful facial expression increased empathy and perceived familiarity. In this study, we measured emotional arousal, familiarity, empathy, and favorability to participants while presenting face stimuli exhibiting six basic emotional expressions (i.e., neutral, joy, surprise, anger, fear, and sadness) under three conditions (i.e., static image, image with asynchronous breathing, and image with synchronized breathing in relation to the participant). In the synchronized condition, a high-precision millimeter-wave radar system was used to capture participants’ real-time breathing patterns, which allowed non-perceptible respiratory monitoring. Comparisons between emotional expressions revealed that the synchronized breathing condition with joyful stimuli was associated with the highest empathy and familiarity, which suggested that a physiological mechanism underlying shared breathing patterns facilitated emotional connection with joyful expressions. These findings offer insights into the embodied basis of positive emotional sharing underlying synchronized breathing during social activities, such as group singing and shared laughter.

**Supplementary Information:**

The online version contains supplementary material available at 10.1038/s41598-026-34981-0.

## Introduction

Empathy refers to a person’s ability to attune themselves to the feelings and sensations of others and plays an important role in daily social interactions^1^. This interpersonal emotional sharing allows individuals to share in others’ happiness and alleviate feelings of sadness. Empathy operates at both psychological and physiological levels, as demonstrated by the synchronization of physiological responses during emotional sharing^2^. Previous studies have shown that observers unconsciously mirror others’ physiological states, such as facial muscle activity^3,4^, cardiac responses, and autonomic nervous system activity^2,5,6^. These findings indicate that empathy emerges from both psychological sharing and physiological linkage. When an observer mirrors another person’s bodily state—such as facial activity, cardiovascular changes, autonomic responses, and even respiration—they create a shared physiological condition. Such synchronization is believed to represent the bodily substrate that underlies empathic experience.

Respiratory mirroring has also been reported previously. In our previous work, we showed that when an individual observes another person holding their breath, they experience increased breathlessness and respiratory frequency (fR)^7^. Moreover, observers’ responses were even stronger when the target person exhibited rapid, deep breathing after the breakpoint. Therefore, these findings suggest that sharing similar breathing patterns enables individuals to empathize with others’ internal states and highlight a potentially unique role of respiration in empathy.

The relationship between breathing and emotion has been studied extensively at the neurophysiological level^8^. Breathing rhythm is regulated by the brainstem, and the primary inspiratory rhythm generator is located in the ventrolateral medulla pre-Bötzinger complex^9^. Emotional states and breathing have a close bidirectional relationship: fear and anxiety are typically accompanied by rapid, shallow breathing, whereas slow, deep breathing promotes pleasantness and comfort^10,11^. Such changes in breathing are associated with activation of and interactions between higher limbic and frontal regions^11^. Therefore, in addition to its homeostatic function, breathing reflects a person’s internal state and can serve as a measurable marker of emotion.

In daily life, expressions related to breathing are often used to describe emotional experiences, such as “to catch one’s breath,” “breathtaking,” and “to take a breath.” Likewise, phrases such as “to be on the same wavelength” and “to be in sync with” describe the process of empathizing with others. These examples illustrate that breathing not only reflects emotion but also plays a role in communication and social connectedness. Beyond these associations, a substantial body of research has demonstrated the broader role played by synchrony in social relationships. Motor synchrony (e.g., coordinated movement), physiological synchrony (e.g., heart rate [HR] coupling), and neural synchrony have each been shown to foster affiliation, trust, and cooperation^12,13^. These findings suggest that synchrony is a general mechanism for strengthening interpersonal bonds. Building on this framework, the present study focused specifically on breathing synchrony, which we consider a distinct and underexplored physiological channel for promoting empathy.

In the current study, we examined whether synchronizing a study participants’s breathing with emotionally expressive face stimuli modulates various outcome variables. Specifically, we assessed (1) emotional state, defined as subjective affective arousal and valence in response to stimuli; (2) empathy, defined as the self-reported sharing and understanding of the depicted expressions; (3) perceived familiarity, defined as the subjective sense of recognition or closeness toward the presented faces; and (4) favorability, defined as the degree of positive evaluation of the faces. To achieve our objectives, we used stimuli depicting seven emotional facial expressions: neutral, joy, surprise, anger, fear, sadness, and disgust^14^. Each image was presented under three conditions: a static image (static condition), an animated image with breathing-like movements that were not synchronized to the observer’s breathing (asynchronous condition), and an animated image with breathing-like movements synchronized to the observer’s breathing (synchronous condition). To achieve precise respiratory synchronization while avoiding discomfort or awareness of measurement, we used a high-precision millimeter-wave radar system for non-perceptible respiratory monitoring. We then examined how breathing synchrony with emotional expressions affects observers’ subjective ratings. In addition, HR was measured to determine whether observed effects were specific to respiration or instead reflected broader autonomic changes. Because HR responses to emotional expressions can influence empathy, we aimed to confirm that the effects of breathing synchrony on empathy were not confounded by parallel changes in HR. Based on previous findings of cardiac mirroring during emotional sharing^2^, we hypothesized that breathing synchrony would specifically enhance empathy as well as social judgements of familiarity and favorability.

## Results

**Fig. 1:**
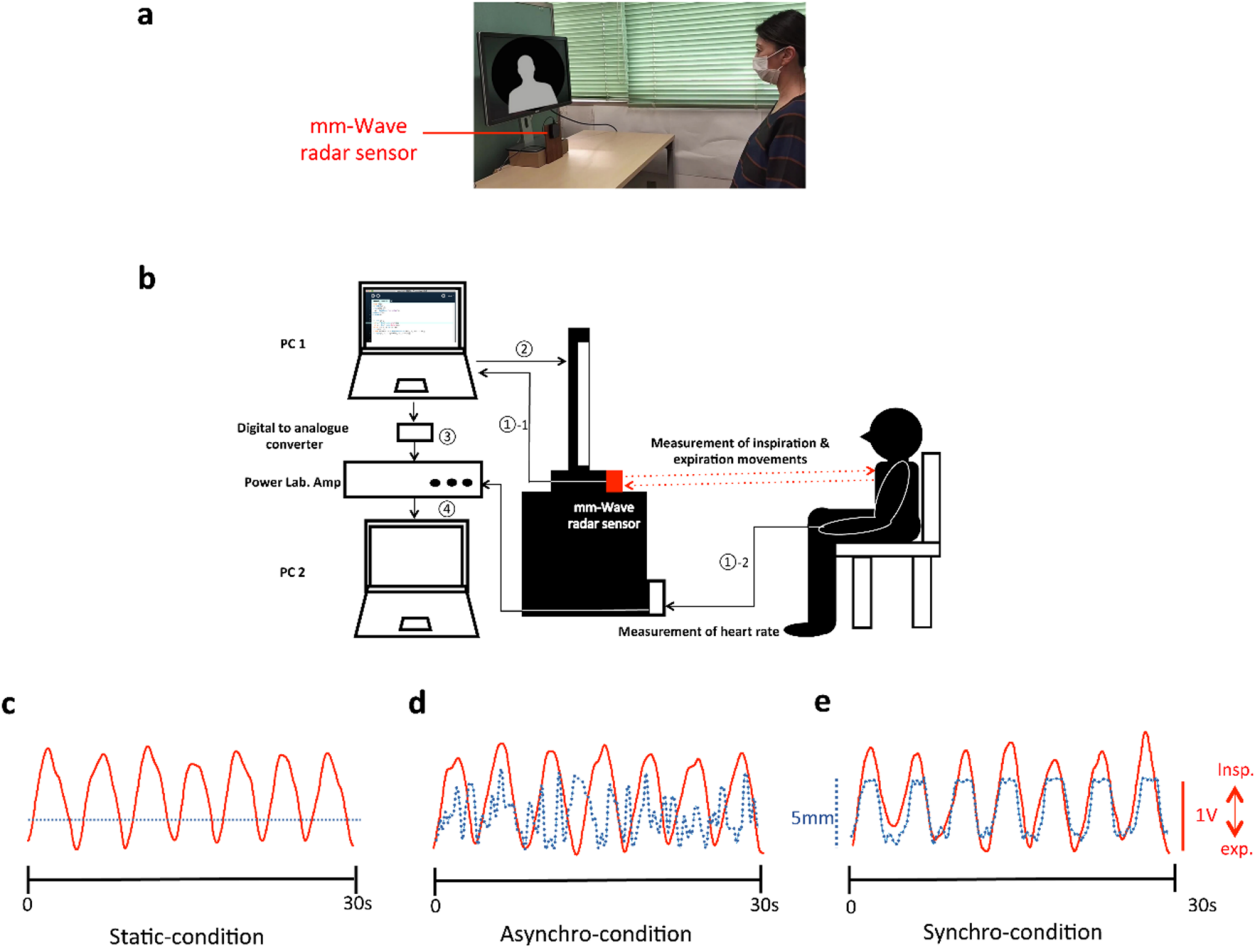
(**a**) Experimental procedure. (**b**) Participants were seated in a chair in front of the screen and the millimeter-wave-radar system. ①-1. The millimeter-wave radar system measured participants’ breathing movement, and the digital data were sent to PC1. ①-2. Heart rate was measured using a photoplethysmogram transducer on the study participant’s left index finger. ② On PC1, the animation matching the study participant’s breathing from the millimeter-wave radar system was created using the processing software and then displayed on the screen. ③ In parallel, digitized respiration data converted into analog format was sent to PowerLab. ④ Respiration and heart rate were simultaneously recorded on PC2 via PowerLab. (**c**–**e**) Representative respiratory movements measured with the millimeter-wave radar (red lines) and vertical movements of the facial expression animation (blue dotted line). Graphs show data extracted over a 30-second period for each condition. (**c**) Static condition. (**d**) Asynchronous (Asynchro) condition. (**e**) Synchronous (Synchro) condition. Facial expression stimuli appearing on the monitor have been replaced with non-identifiable schematic illustrations for display purposes.

We tested the effects of breathing synchronization on empathy using a millimeter-wave radar system to synchronize study participants’ breathing with facial expression stimuli in real time (Fig. 1a,b, and Supplementary Methods online). We tested three conditions for seven emotional facial expressions: static condition (Fig. 1c, and Supplementary Video S1 online), asynchronous condition (Fig. 1d and Supplementary Video S2 on line), and synchronous condition (Fig. 1e, and Supplementary Video S3online). A total of 84 images comprising seven emotion types were presented. Each emotion type was tested for all three conditions (i.e., static, synchronous, and asynchronous), and two images (one female and one male face) were presented for each expression category to minimize the effects of implicit sex-related and preference biases. To evaluate the identification of emotions, subjective scores for emotional arousal, familiarity, empathy, and favorability were obtained after presenting the image for 30 s. Respiration and cardiac output were continuously measured throughout the experiment. Subjective scores for disgust stimuli were excluded from the statistical analysis because of the low accuracy rate for judging the facial expression of disgust (the accuracy rate was 42.7%, and most participants identified disgust as fear or anger). Scores with an accuracy rate of over 70% were used for subsequent analyses (accuracy rates were as follows: neutral, 84.1%; joy, 95%; surprise, 94%; anger, 85.5%; fear, 75.9%; and sadness, 91.7%). To compare the subjective scores between the four image types and examine whether the subjective scores for the four images were influenced by participants’ sex, type of emotion, and condition, we performed a four-way analysis of variance (ANOVA), with subjective score type as the dependent variable and emotion type, condition, and sex as independent variables. There were no significant differences in subjective scores between the four images (two female images and two male images), and there was no significant interaction between emotion type, condition, and sex (Supplementary Results online). Therefore, subjective scores were averaged, and one score was calculated for each condition for each emotion type.

**Fig. 2:**
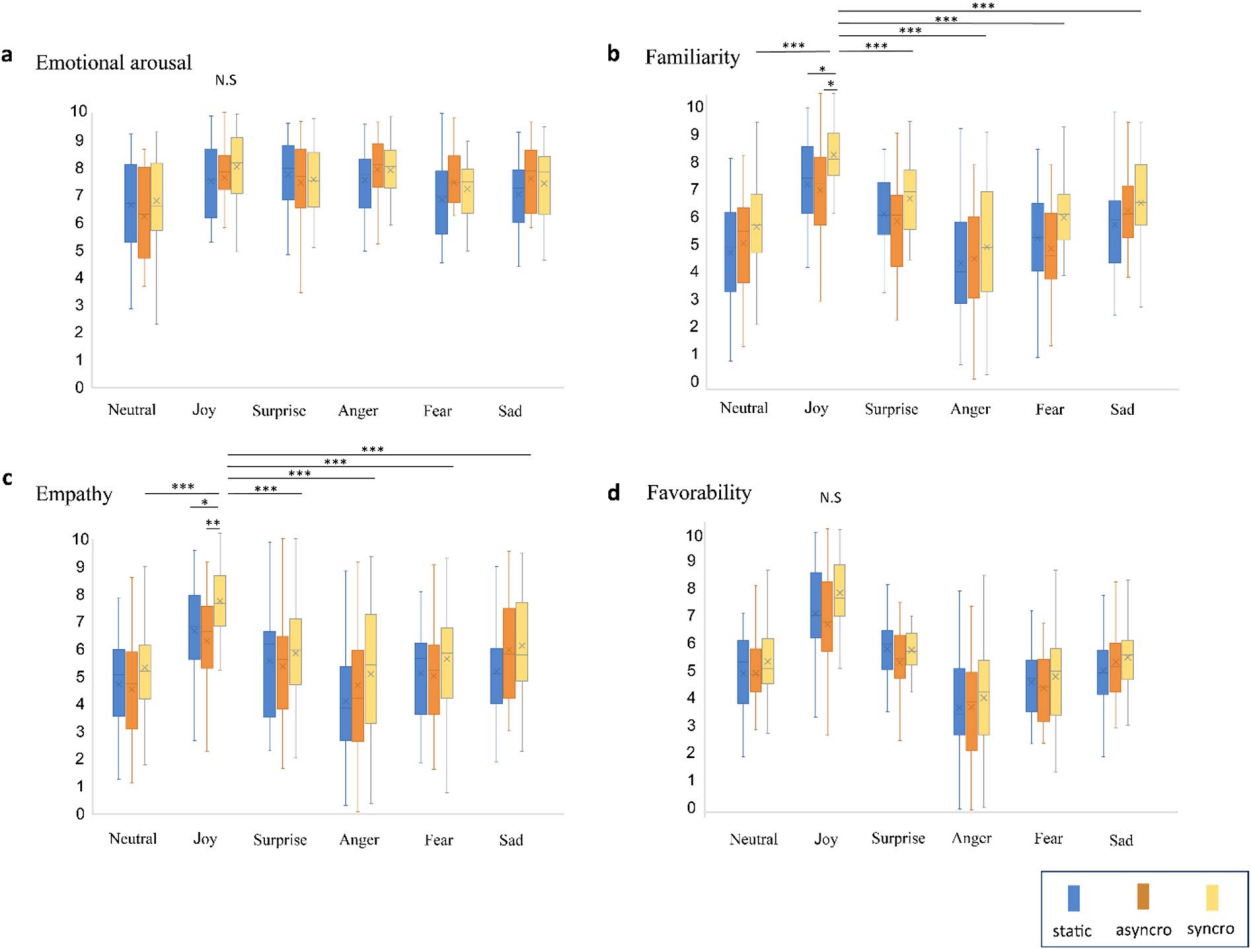
Results of emotional arousal (**a**), familiarity (**b**), empathy (**c**), and (**d**) favorability scores. Horizontal lines and cross marks of each box indicate the mean and median values of the group dataset. Familiarity and empathy scores for joyful stimuli were higher in the synchronous (synchro) condition than those in the static and asynchronous (asynchro) conditions. Joyful stimuli in the syncro condition yielded the highest scores out of all emotion types and conditions. There were no main effects for emotional arousal or favorability. *p < 0.05, **p < 0.001, ***p < 0.0001.

The effects of emotion type and condition on emotional arousal (Fig. 2a), familiarity (Fig. 2b), empathy (Fig. 2c), and favorability (Fig. 2d) are presented. Statistical analysis results were presented in Table 1. Statistical reports of interaction follow-up analysis were presented in the Supplementary Information (Supplementary Tables S1-S24 online). The two-way ANOVA revealed significant main effects of emotion type (F_5, 546_ = 15.32, *p* < 0.0001, *η*^2^ = 0.12) and condition (F_2, 546_ = 8.3, *p* = 0.0001, *η*^2^ = 0.03) on empathy. Comparison between types of emotion and empathy revealed higher scores for the joyful stimuli than for other types of emotion (all *p* < 0.0001, except for joy vs. sadness for empathy scores, which was *p* = 0.003). There were significant difference between static and synchro conditions (*P* = 0.0001), and between asynchro and synchro condition (*P* = 0.005).

**Table 1:** Statistical analysis results of two-way ANOVA with subjective score (emotional arousal, empathy, familiarity, and favorability) as the dependent variable, and the types of emotion (six types) and condition (three conditions) as independent variables.

	Main effects								Interaction			
	Emotion type				Condition				Emotion type * Condition			
	df	F	Sig.	ηp2	df	F	Sig.	ηp2	df	F	Sig.	ηp2
Emotional arousal	5, 551	11.25	0.0001	0.09	2, 551	1.92	0.14	0.007	10, 551	1.1	0.35	0.02
Familiarity	5, 549	28.02	0.0001	0.2	2, 549	10.41	0.0001	0.03	10, 549	0.63	0.78	0.01
Empathy	5, 546	15.32	0.0001	0.12	2, 546	8.3	0.0001	0.03	10, 546	0.76	0.66	0.01
Favorability	5, 550	50.02	0.0001	0.31	2, 550	4.99	0.007	0.02	10, 550	0.58	0.83	0.01

Statistical reports of interaction follow-up analysis were presented in the Supplementary Tables S1-S24.

The synchronous condition yielded higher empathy for joyful stimuli than the static (joyful stimuli: F_2,546_=5.479, *P* = 0.004, post hoc comparison, *p* = 0.020) and asynchronous conditions (*p* = 0.009). Empathy scores in the synchronous condition were highest for the joyful stimuli than for other emotion types (synchro condition: F_5,546_ = 7.861, *P* = 0.0001; post hoc comparisons; joy vs. neutral: *p* = 0.0001; joy vs. surprise: *p* = 0.001; joy vs. anger: *p* = 0.0001; joy vs. fear: *p* = 0.0001; and joy vs. sadness: *p* = 0.013).

Familiarity scores showed a similar pattern to empathy scores. There were significant main effects of emotion type (F_5, 549_ = 28.02, *p* < 0.0001, *η*^2^ = 0.2) and condition (F_2, 549_ = 10.41, *p* < 0.0001, *η*^2^ = 0.03) on familiarity. Multiple comparisons revealed that familiarity scores for joyful stimuli were significantly higher than those for other emotion types (all *p* < 0.0001). There were significant difference between static and synchro conditions (*P* = 0.0001), and between asynchro and synchro condition (*P* = 0.0001).

Familiarity scores were significantly higher in the synchronous condition than in the static (joyful stimuli: F_2, 549_=4.613, *P* = 0.01; post hoc comparison, *p* = 0.05) and asynchronous conditions (*p* = 0.01). Familiarity scores in the synchronous condition for joyful stimuli were significantly higher than those for other emotion types (syncro condition: F_5, 549_=12.49, *p* = 0.0001; post hoc comparisons; joy vs. neutral, joy vs. anger, and joy vs. fear: *p* = 0.001; joy vs. sad: *p* = 0.002; and joy vs. surprise: *p* = 0.008). We also revealed a significant main effect of emotion type on emotional arousal (F_1, 551_ = 11.25, *p* < 0.0001, *η*^2^ = 0.09) but there was no significant main effect of condition on emotional arousal (F2,551 = 1.92, *P* = 0.14, *η*^2^ = 0.007 ). Post hoc comparisons revealed overall emotional arousal scores for joy, surprise, anger, fear, and sadness stimuli were higher than those for neutral images (*p* < 0.05 for all pairs).

There was a significant main effect of emotion type on favorability scores (F_5,550_ = 50.02, *p* < 0.0001, *η*^2^ = 0.31) but no significant effect of condition on favorability scores (all *p* > 0.05). Comparison between types of emotion and favorability revealed higher scores for the joyful stimuli than for other types of emotion (all *p* < 0.0001). Also, favorability scores for joyful stimuli in each condition were higher than those for all other emotion types (static condition: F_5,550_=17.11, *p* = 0.0001, *η*^2^ = 0.13; asynchor condition: F_5, 550_ = 13.19, *p* = 0.0001, *η*^2^ = 0.11; synchro condition: F_5, 550_ = 20.8, *p* = 0.0001, *η*^2^ = 0.15; pot hoc comparison, all *p* < 0.0001). However, there were no significant difference in favorability between static and synchro conditions (*P* = 0.94), and between static and asynchro condition (*P* = 0.18). Significant results were consistent across false discovery rate (FDR) corrections indicated in the Supplementary Tables S1-S24 (online).

**Fig. 3:**
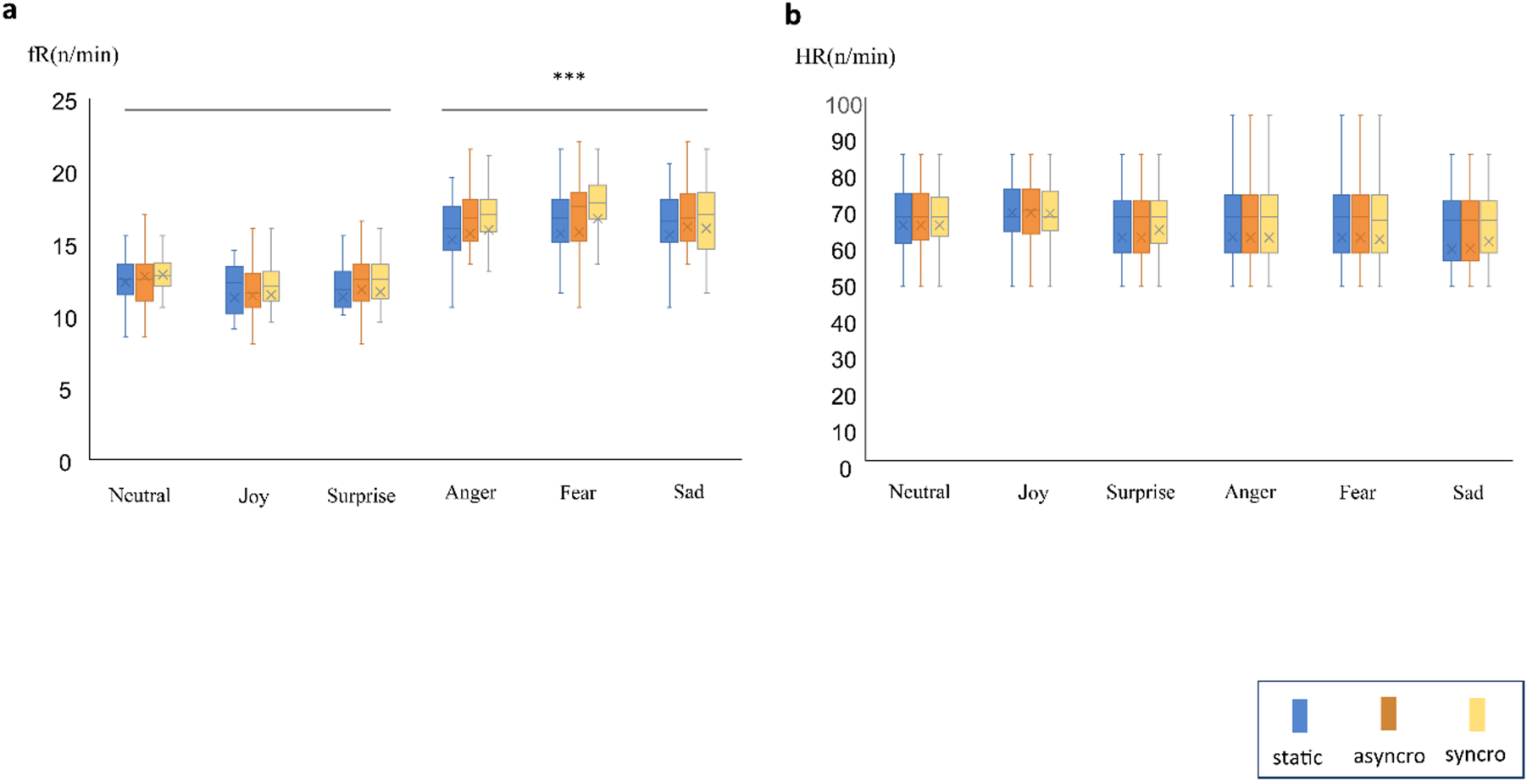
The results of the (**a**) respiratory frequency (fR) and (**b**) heart rate (HR) analyses. Data are represented as numbers for fR and per minute for HR. Horizontal lines and cross marks in each box indicate the mean and median values of the group dataset. FR was higher for anger, fear, and sadness than for neutral, joy, and surprise stimuli. There were no differences in fR among the three conditions for any emotion type. HR remained constant across all emotion types and conditions. Asynchro, asynchronous; syncro, synchronous. ***p < 0.0001.

The physiological data (Fig. 3) revealed a main effect of emotion type on fR (F_5,552_ = 135.17, *p* < 0.0001, *η*^2^ = 0.56)(Fig. 3a) but not HR (F_5,510_ = 0.68, *p* = 0.63, *η*^2^ = 0.007; Fig. 3b) (Table 2). Multiple comparisons showed that respiratory rate increased more during the presentation of anger, fear, and sadness stimuli than during the presentation of neutral, joy, and surprise stimuli (all *p* < 0.0001). There were no significant differences in fR among anger, fear, and sadness stimuli (anger vs. sadness: *p* = 0.45 and *p* = 1 for other pairs). There were no significant differences in fR among neutral, joy, and surprise stimuli (all *p* = 1). There was a significant main effect of condition on fR (F_2.552_ = 4.23, *p* < 0.01, *η*^2^ = 0.16) but not HR (F_2.510_ = 1.61, *p* = 0.2, *η*^2^ = 0.006). There was no significant emotion type * condition interaction for either fR (F_10,522_ = 0.2, *p* = 0.99, *η*^2^ = 0.004) or HR (F_10,510_ = 0.68, *p* = 0.73, *η*^2^ = 0.01). Statistical reports of interaction follow-up analysis were presented (see Supplementary Table S25-S32 online). The physiological data analysis showed that HR was not affected by the type of emotion or condition. However, fR was significantly affected by the type of emotion, especially negative emotions. Condition did not significantly affect fR for any emotion type.

**Table 2:** Main effects of emotion and condition and the emotion × condition interaction for respiratory frequency (fR) and heart rate (HR).

	Main effects								Interaction			
	Emotion type				Condition				Emotion type * Condition			
	df	F	Sig.	ηp2	df	F	Sig.	ηp2	df	F	Sig.	ηp2
fR	5, 552	135.17	0.0001	0.56	2, 522	4.23	0.01	0.16	10, 522	0.2	0.99	0.004
HR	5, 510	0.68	0.63	0.007	2, 510	1.61	0.2	0.006	10, 510	0.68	0.73	0.01

Statistical reports of interaction follow-up analysis were presented in the Supplementary Tables S25-S32.

**Fig. 4:**
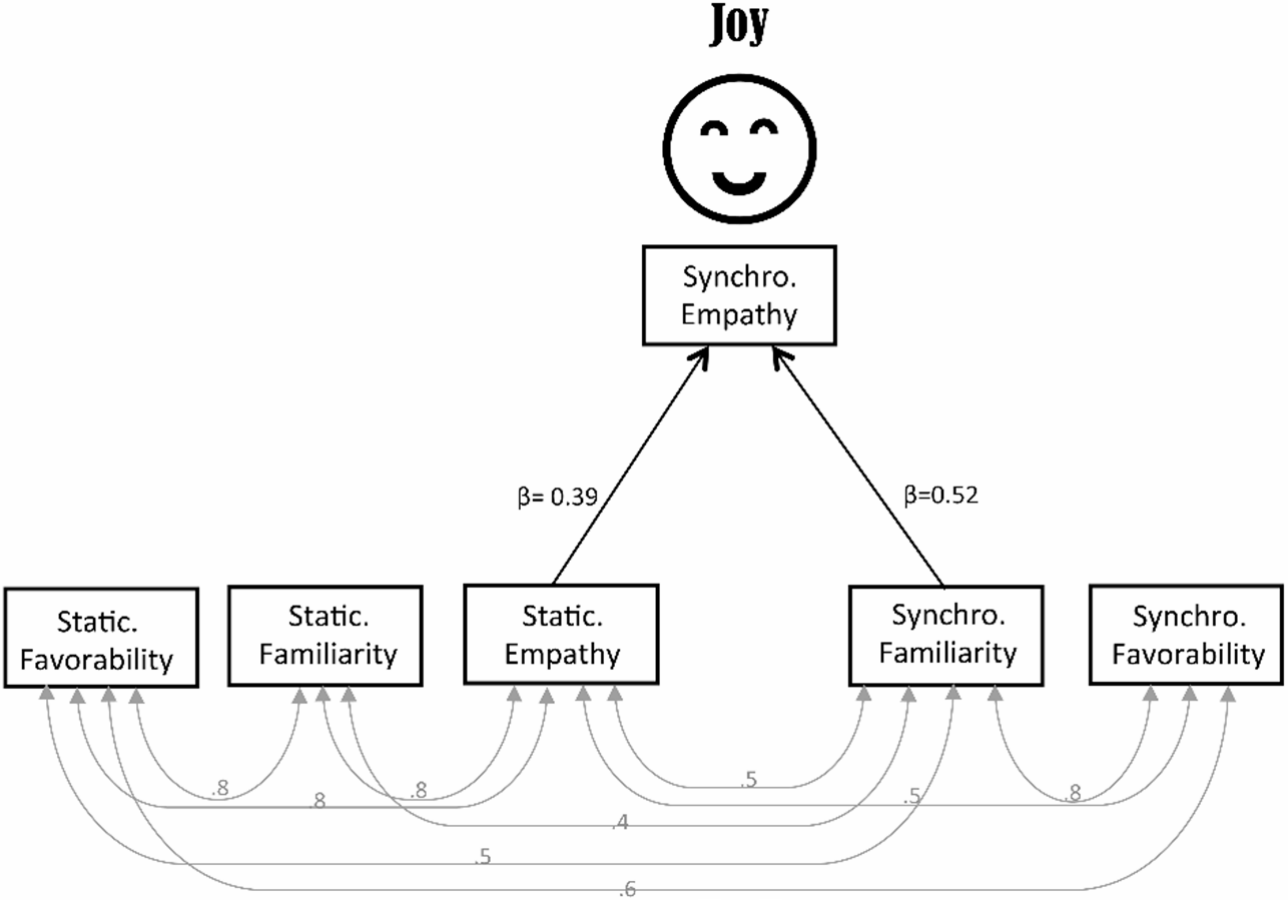
Exploratory path analysis examining relationships between empathy and familiarity for joyful stimuli, and between synchronous (synchro) and static conditions. Non-significant paths involving the asynchronous condition were excluded for clarity. Straight arrows represent standardized beta coefficients, and double-headed curved arrows represent correlations. Although the primary ANOVAs did not yield significant interaction effects, this model was explored to investigate potential mediating links (e.g., familiarity → empathy). Findings should be considered exploratory and hypothesis-generating. The smile-like image shown in Fig. 4 is a schematic illustration created by the author for display purposes.

To further explore potential relationship among the outcome variables beyond the ANOVA results, we conducted an exploratory path analysis to examine possible mediating relationships among the dependent variables. Specifically, we tested whether the effect of synchronized breathing on empathy could be explained, in part, by an increase in familiarity. This approach was motivated by previous evidence linking familiarity and empathy^1,5,15^ and the significant ANOVA findings for these variables. Therefore, the path analysis was intended to complement, rather than replace, the ANOVA results, and the findings should be interpreted as preliminary and hypothesis-generating. The final path model (Fig. 4) was constructed by eliminating non-significant paths. Because there were no significant paths for the asynchronous condition, all paths connected to the asynchronous condition were excluded. The model fit was confirmed by a goodness of fit index of 0.99 and Bollen–Stine bootstrapping (*p* = 0.97). Empathy for joyful stimuli in the synchronous condition was significantly affected by familiarity for joyful stimuli (*β* = 0.52, *p* < 0.0001); furthermore, familiarity for joyful stimuli was associated with favorability (*β* = 0.8, *p* < 0.0001; Fig. 4). Empathy in the synchronous condition showed a higher score than that in the static condition (Fig. 2c), although empathy in the static condition significantly affected empathy in the synchronous condition (*β* = 0.4, *p* = 0.004). In both the static and synchronous conditions, there was an interrelationship between empathy, familiarity, and favorability (indicated by gray double-headed arrows and r-values in Fig. 4). This finding indicates that empathy scores for joyful stimuli were already high during the static condition and that synchronized breathing further increased empathy scores for joyful stimuli.

## Discussion

In this study, we examined whether viewing animated facial expressions with breathing movements synchronized to the observer’s own respiration influences emotional arousal, empathy, familiarity, and favorability. We found that synchrony selectively enhanced empathy and familiarity for joyful expressions, which was not observed in the static and asynchronous conditions. In the synchronized breathing condition, empathy and familiarity scores for joyful stimuli were higher than those for any other emotion type. These effects persisted even after controlling for sex differences indicated in the Supplemtary Information (Supplementary Fig. S1). Importantly, these effects of synchrony were specific: breathing synchronization enhanced empathy and familiarity but did not significantly influence emotional arousal or favorability, which were instead modulated by emotion type. This suggests that empathy and familiarity are particularly sensitive to bodily synchrony, whereas arousal and favorability are more directly dependent on the valence and intensity of expressions.

Exploratory path analysis indicated a possible association between familiarity and empathy for joyful faces, which aligned with previous reports that familiarity enhances empathy^1,15,16^. Even in the static condition, empathy for joyful faces was associated with high familiarity and favorability, and effect was further amplified by synchrony. This may be because aligning breathing with observed expressions allows individuals to perceive their own physiological state, which in turn strengthens the match between emotion and respiration, and in turn, enhances both familiarity and empathy.

An alternative explanation is that changes in an observer’s facial muscle activity contribute to an increase in empathy and familiarity for joyful stimuli. A previous study reported that facial muscle activity during the observation of joyful expressions is associated with heightened feelings of pleasantness and comfort^17^. In our study, such muscle activity may have enhanced empathy for joyful faces across all conditions, with the strongest effect induced by the synchronized breathing condition. Visually matched breathing movements may have further amplified this process by reinforcing facial muscle tension linked to joyful expressions.

Emotional experience is closely tied to physiological responses, such as facial mimicry^3,4,18^, autonomic changes^19^, and neural activation^20,21^. Respiration is also associated with internal emotional states and is linked to limbic activity, especially that of the amygdala^11^, which plays a central role in processing facial expressions^21^. Parallel processing of facial expression recognition and respiratory changes may thus occur during evaluation of emotional stimuli via amygdala activation. We initially speculated that synchronization of higher breathing frequencies associated with negative emotions, together with amygdala activity, would more effectively promote empathy for aversive expressions. However, we found that synchrony did not increase empathy for negative emotions. One possible explanation for this observation is a ceiling effect: empathy for negative emotions may have already been high, which left limited capacity for further enhancement. Another possibility is that participants downregulated their responses to aversive expressions, thereby reducing the influence of synchrony.

In contrast, joy seemed to be uniquely sensitive to slower breathing synchrony. Activities related to positive emotions, such as laughter, singing, and group breathing, often rely on shared physiological states^22–24^ that foster bonding and reward^25^. Meta-analytic evidence indicates that behavioral and physiological synchrony are consistently associated with positive emotions and experiences of self–other overlap, paralleling the enhanced familiarity observed in our study^26^. Therefore, synchrony may be particularly important for amplifying joy, whereas negative emotions may elicit stronger activity than positive experiences in empathy-related brain regions^27^, regardless of synchrony. This interpretation aligns with the “undoing hypothesis,” which suggests that positive emotions help reduce physiological arousal induced by negative states^28^. Additionally, breathing synchrony may contribute to this process by reinforcing the regulatory effect of joy, thereby facilitating a reduction in tension^29,30^. Thus, breathing synchrony in joyful contexts may provide a dual reward: enhancing pleasantness without increasing negative affect.

Another perspective of our findings involves distinguishing empathy from emotional contagion. Emotional contagion refers to the automatic “catching” of another person’s emotional state^31^, whereas empathy is defined by both affective sharing and an awareness that the emotion belongs to the other person^32^. It is possible that the heightened responses to synchronized joyful breathing in our study primarily reflect emotional contagion rather than higher-order empathy. Nevertheless, such automatic resonance may be the physiological mechanism underlying more complex empathic processes. Future research combining physiological, behavioral, and neural measures will be needed to disentangle these mechanisms.

Several limitations should be acknowledged. First, disgust stimuli were excluded because of low recognition accuracy, which is consistent with previous evidence showing that the identification of disgust is more difficult than the identification of other emotions^33,34^. Excluding one of the six basic emotions limits the generalizability of our findings across the entire emotional spectrum. Second, HR did not show significant modulation in any condition, which was likely due to the relatively short stimulus presentation duration that was insufficient to evoke reliable cardiac changes. Longer exposure times or sustained emotional stimuli may induce a stronger effect. Third, although we provided operational definitions of the outcome measures, constructs such as empathy, familiarity, and favorability remain broad. Participants may have interpreted these differently, which introduced variability into ratings. Using standardized scales and providing more detailed examples may improve consistency in future research. Fourth, although participants were not told about the synchrony manipulation, some may have perceived alignment between their breathing and the stimuli’s movements. Such awareness could have influenced the subjective ratings or physiological responses. Future studies should assess participants’ awareness of synchrony more directly. Fifth, respiration was measured by frequency only. Other variables, such as tidal volume, inspiration and expiration time, and functional residual capacity, were not captured by the millimeter-wave radar system. However, these parameters may also play important roles in emotional breathing patterns and should be included in future work. Sixth, the sample size was relatively small (*N* = 32). Given *N* = 32, some smaller effects may have been undetected under strict Bonferroni control. We mitigated this by reporting uncorrected, Bonferroni-, and FDR-corrected results for transparency, and we present the current findings—especially the synchrony-related enhancement for joy—as initial evidence to be replicated in larger samples. Sixth, we did not measure participants’ facial muscle activity. Facial muscle responses may partly underlie the enhanced empathy and familiarity observed for joyful stimuli. Future studies should include facial electromyography to clarify this possibility.

In addition to these experimental constraints, previous studies have linked empathy to widespread neural systems, such as mirror neurons and the insula, which have also been implicated in respiration^19–21,25,35,36^. Investigating brain activation and connectivity during breathing synchrony would provide valuable insights into the underlying mechanisms. Finally, the present study was conducted in a laboratory using unfamiliar faces. In real-world interactions, synchrony with familiar individuals may produce stronger or qualitatively different effects. Therefore, replication in naturalistic settings will be essential.

In conclusion, synchronized breathing with joyful expressions enhanced both empathy and familiarity. These findings provide initial evidence that shared respiratory rhythms strengthen positive emotional connections, which supports the broader view of synchrony as a mechanism for social bonding. Our results also resonate with everyday social behaviors; singing, chanting, and laughter are synchronous activities that rely on breathing and foster affiliation. Therefore, respiratory synchrony may represent one pathway through which such collective activities strengthen social bonds.

## Methods

### Participants

This study was approved by the ethics committee of Showa University School of Medicine and conducted according to the principles of the Declaration of Helsinki (trial identifier number: 3,327). An analysis using G*Power (Version 3.1.9; University of Dusseldorf, Dusseldorf, Germany) indicated that a sample size of 32 would be expected to have statistical power of 95% for detecting a medium effect with an alpha value of 0.05. The effect size (0.2) was determined by referring to previous studies on facial expression processing^29^. However, we note that this estimate does not fully account for the increased stringency introduced by multiple comparison correction. Thus, the actual power to detect smaller effects may be lower. A total of 32 participants (mean age: 30.19 years; standard deviation: 9.46; age range: 20–50 years; 20 female participants, 12 male participants, all right-hand dominant) participated in this study. All participants provided written informed consent before the study and were paid 3,000 yen for their participation. None of the participants had any history of psychological or neurological disease, and had normal or corrected-to-normal vision. All participants were friends of the authors or university staff who were not familiar with medicine or psychology. All participants were asked to remove all metal attachments (e.g., jewellery, buttons, and watches) before the study, and all wore face masks to reduce the risk of infection. The mean level of education was 17.6 ± 3.4 years. Before the experiment, all participants were assessed using Spielberger’s State and Trait Anxiety Inventory (state score: 38.06 ± 5.7; trait score: 44 ± 5) and the Center for Epidemiologic Studies Depression Scale (CES-D)(33 ± 8.22) to confirm there was no presence of anxiety or depression. In the Fig. 1 and Supplementary Videos S1, S2 and S3 (online), one of the authors appears from the back and the side. Written informed consent for the use of these materials in an online-access publication was obtained from the author.

### Apparatus

The experimental apparatus is shown in Fig. 1. After participants provided informed consent and completed assessments, participants were seated in a chair positioned 1 m in front of a 27-inch screen. We used color images of emotional faces obtained from the ATR Facial Expression Image Database (DB99, ATR-Promotions, Kyoto, Japan; http://www.atr-p.com/face-db.html) as the stimuli. Color images of emotional faces were obtained from the ATR Facial Expression Image Database. The facial expression images originally presented on the monitor were removed and replaced with non-identifiable schematic illustrations for display purposes in Fig. 1 and the Supplementary Video S1-S3.

A total of 84 facial images were presented, which comprised seven emotional expression types under three conditions. For each expression type, two female (F1 and F2) and two male (M1 and M2) images were used. Stimuli were presented in randomized order to minimize identity-related bias, and a blank screen with a fixation dot at eye level was interspersed between each image. Randomization was performed using the Microsoft Excel RAND function, and stimuli were presented manually by three researchers in turn according to the randomized sheet. Each image was displayed for 30 s to avoid adaptation of respiratory responses to emotional state. This is because 30 s is considered the maximum duration for maintaining a stable carbon dioxide level in the body while evoking emotional arousal^37,38^. Participants were instructed to breathe normally while viewing the face stimuli. They were not informed that the stimuli’s breathing movements may be synchronized with their own respiration, and were not asked to deliberately control their breathing or the animation timing. Before the experiment, a 5-min resting-state measurement of fR was obtained to establish each participant’s baseline breathing pattern, which served as a reference for stabilization between trials. Immediately after each 30-s stimulus presentation, participants identified the expressed emotion by selecting one of seven emotion types. Emotional arousal, familiarity, empathy, and favorability were then rated on a visual analogue scale.

To ensure participants’ understanding of the outcome variables was consistent, we provided standardized explanations of each outcome variable before the experiment. Specifically, we informed participants that (1) emotional state refers to subjective affective arousal and valence in response to the stimuli; (2) empathy refers to emotional sharing with and understanding of the depicted expressions; (3) perceived familiarity refers to recognition or closeness toward the presented faces; and (4) favorability refers to the degree of positive evaluation or liking assigned to the stimuli. These explanations were provided both verbally and in written form before the rating task. The visual analogue scale consisted of a 10-cm horizontal line, with the left end representing “not at all” and the right end representing “very much.” To assess emotional arousal, participants reported the intensity of arousal they felt. For familiarity and empathy, they indicated the degree of familiarity and empathy they experienced in relation to the stimuli. For favorability, they rated how positively they evaluated the stimuli. After each rating, we confirmed that participants’ breathing had returned to baseline before presenting the next stimulus.

Respiration was measured using a millimeter-wave radar system (see Experimental conditions in the Method, and Supplementary Methods, Supplemetary Table S33 and Supplemetary Fig. S2 online for details). Data were converted from digital to analogue via a converter and stored in LabChart via PowerLab (ML846; ADInstruments, Aichi, Japan). The accuracy of respiratory measurements was confirmed by simultaneously recording chest wall movement using a belt sensor (TN 1132/ST, AD Instruments, Aichi, Japan; Supplementary Fig. S3). FR was computed in LabChart by peak detection of inspiratory cycles. HR was recorded with a photoplethysmogram transducer placed on the left index finger (TSD200-MRI and PPG100C-MRI; BioPac, LA System, Japan), and signals were stored in LabChart via PowerLab (Lenovo, IBM, Japan). HR was extracted by detecting successive pulse peaks. Both fR and HR were sampled at 1000 Hz. During acquisition, a 50-Hz low-pass filter was applied to reduce high-frequency noise. For analysis, a digital 10-Hz low-pass filter was applied within LabChart before peak detection. No additional preprocessing was performed.

### Experimental conditions

Condition 1 was the static condition, in which each image was static (no breathing movement) for each facial expression (Supplementary Video 1). Condition 2 was the asynchronous condition, in which the breathing-like movement in the image was random and smooth, as determined using the Perlin noise function (Supplementary Video 2). Condition 3 was the synchronous condition, in which the breathing-like movement in the image was synchronized with the study participant’s respiration (Supplementary Video 3). To synchronize the movement of the stimuli with the study participant’s breathing (inhalation and exhalation), we used a millimeter-wave radar system (RFR79ITR34-30U, PTM Corp., Yokohama) to detect the study participant’s respiratory activity. Details of the millimeter-wave radar are reported in the Supplementary Methods, Supplementary Table S33 and Supprementary Fig. S2 online.

Briefly, the millimeter-wave radar uses radio waves in the 30–300 GHz range, which corresponds to wavelengths of 1–10 millimeters. This frequency range is higher than that of traditional radar systems that operate in the microwave frequency range. One of the key advantages of millimeter-wave radar is its ability to provide high-resolution imaging and sensing. Millimeter-wave radar has been applied to automotive radar. This short wavelength of millimeter-wave systems enables the detection of the study participant’s body movement in the order of millimeters. Measurements of inhalation- and exhalation-related chest movements were digitized immediately and transferred to a computer. Animations of the body movement of the facial image were created for each emotion type using Adobe Photoshop (Adobe Inc., USA). Each frame in the animation was a still image that was slightly modified from the previous image. A total of 120 still images were created for each emotion type, which were played in sequence to generate an illusion of movement. In these frames, the face remained static, but the movements of the shoulders and arms were depicted in sequence as if they were synchronized with inspiration and expiration. To match this animation to the study participant’s breathing digitized via the millimeter-wave radar, we used the Processing (https://processing.org/) programming language. The digitized inhalation and exhalation movement data were input into a computer to control the animated movement as if the person depicted in the stimuli exhibited synchronized breathing with the observer (Videos S3). To confirm the accuracy of the match in breathing movements between the animation and the participant, we simultaneously filmed the movement of both and digitized the respiratory movement using the Kinovea software (https://www.kinovea.org). Both types of tracked data were exported as a comma-separated value file. Similarity was confirmed via a cross-correlation analysis (*r* = 0.8).

### Statistical analysis

All tests were performed using SPSS (SPSS version 23, IBM Corp., Armonk, NY, USA).

A four-way ANOVA was performed, with the type of subjective score (i.e., emotional arousal, empathy, familiarity, or favorability) as the dependent variable, and emotion type (six types), condition (three conditions), and sex as independent variables. Subjective scores were separately analyzed. There was no difference in subjective scores between the four images (i.e., the two female and two male face images)(emotion, F_3,1855_ = 1.89, *p* = 0.15, *η*^2^ = 0.002; empathy, F_3,1876_ = 1.11, *p* = 0.34, *η*^2^ = 0.002; familiarity, F_3,1854_ = 0.36, *p* = 0.78, *η*^2^ = 0.001; favorability, F_3,1854_ = 2.5, *p* = 0.06, *η*^2^ = 0.004) and no significant interactions. Then each study participant’s scores were averaged and expressed as one score for each condition for each emotion type (e.g., emotion level for study participant 1: anger-static, 7.3, anger-asynchronous, 7.4, and anger-synchronous, 7.8). After confirming the absence of any bias noted above and simplifying the data, we analyzed the subjective scores for emotional arousal, empathy, familiarity, and favorability separately using a two-way ANOVA. All data were normally distributed, as assessed using the Shapiro–Wilk test, and had equality of variance, as assessed using Levene’s test (all *p* > 0.05). The two-way ANOVA was performed with subjective score type as the dependent variable, and emotion type and condition as independent variables. The analysis provided omnibus of the main effets and emotion type × condition interaction. To further probe specific differences, we conducted simple effects analysises using estimated marginal measn (EMMEANS) in SPSS. We examined (a) simple main effects of emotion type within each condition (yielding 3 omnibus F tests) and (b) simple main effects of condition within each emotion type (yielding 6 omnibus F tests). Pairwise comparisons were Bonferroni-adjusted to control for multiple testing. For each dependent variable, Bonferroni correction was applied within each family of pairwise comparisons: 15 (emotion main effect), 3 (condition main effect), 45 (emotion within condition), and 18 (condition within emotion); 81 comparisons per dependent variable in total. We did not apply a single correction across all families. All reported *p*-values in the text are Bonferroni corrected. For transparency, we report both Bonferroni corrected and uncorrected p-values were reported. FDR correction was also applied and reported in the Supplementary Tables S1-S24.

For the physiological measurements of fR and HR, normal distributions and equality of variances were confirmed using the Shapiro–Wilk test and Levene’s test, respectively. Two-way ANOVAs were performed with fR or HR as the dependent variable and emotion type and condition as independent variables. Bonferroni post hoc tests were used to correct for multiple comparisons for significant main and interaction effects. All statistical tests were performed using SPSS version 23 (IBM Corp., Armonk, NY, USA).

Path analysis (Amos version 27.0, IBM Corp., Armonk, NY, USA) was used to investigate the interrelationships among empathy, familiarity, and favorability across conditions, and to identify which factors most strongly influenced empathy in the synchronous condition. Unlike hierarchical regression, this approach allows simultaneous modeling of both the direct and indirect effects among variables. The model was theory-driven: we assumed that empathy elicited under static and asynchronous conditions would contribute to empathy under the synchronous condition, reflecting carry-over effects of prior empathic responses. Therefore, we specified the model a priori to test the relative influence of familiarity and favorability on empathy in synchrony, rather than selecting a model via exploratory model fitting.

Statistical significance was set at an adjusted *p* < 0.05. Path analysis estimates were calculated for the magnitude and significance of the presumed causal links among variable sets. Straight arrows linking the variables indicate the direction of causal relationships between variables. Double-headed curved arrows indicate correlations between exogenous variables. Standardized beta coefficients are indicated by straight arrows. Before drawing conclusions from the path analysis results, we calculated several indices to evaluate the validity of the results: the goodness-of-fit index and the Bollen–Stine bootstrap value. A goodness-of-fit index value close to 1 and a Bollen–Stine bootstrap *p* > 0.05 were used to assess the model.

## Supplementary Information

Below is the link to the electronic supplementary material.

Supplementary Material 1

Supplementary Material 2

Supplementary Material 3

Supplementary Material 4

## Data Availability

The datasets used during the current study available from the corresponding author on reasonable request.
