# Supplementary material for: Shared breath of joy enhances empathy through breathing synchronization
Source: Sci Rep. 2026 Jan 5;16:4754. doi: 10.1038/s41598-026-34981-0 (PMC12873253; doi:10.1038/s41598-026-34981-0)
Supplement: Supplementary file 1 — Supplementary Material 1 [file 41598_2026_34981_MOESM1_ESM.doc]

**Shared breath of joy enhances empathy through breathing synchronization**

**Supplementary Information**

Yuri Masaoka^1*^, Motoyasu Honma^1^, Momoka Nakayama^2^, Misako Matsui^1^, Akira Yoshikawa^3^, Shota Kosuge^4^, Miku Kosuge^5^, Daiki Shoji^4^, Shunsuke Sakakura^1^, Masahiko Izumizaki^1^

1. Department of Physiology, Showa Medical University School of Medicine, Tokyo, Japan.
2. Dentsu Lab Tokyo, Tokyo, Japan.
3. Division of Health Science Education, Showa Medical University School of Nursing and Rehabilitation Sciences, Yokohama, Japan.
4. Division of Neurology, Showa Medical University Hospital, Tokyo, Japan.
5. Department of Respiratory Medicine, Showa Medical University Fujigaoka Hospital, Yokohama, Japan.

***Corresponding Author:**

Yuri Masaoka (faustus@med.showa-u.ac.jp)

Department of Physiology, Showa University School of Medicine

1-5-8 Hatanodai, Shinagawa-ku, Tokyo 142-8555, Japan

Tel: +81-3-3784-8113

Fax: +81-3-3784-0200

Figures 1-3

Tables 1-33

Video descriptions 1, 2, 3

**Supplemental Results**

No difference in subjective scores between four image types

To test the difference in subjective scores between four image types and to examine whether subjective scores for these four images were influenced by study participant’s sex, type of emotion, and condition, four-way ANOVA was performed with the type of subjective score as the dependent variable, and emotion type, condition, and sex as independent variables. There were no significant differences of each subjective score between the four image types (emotion, F_3,1855_ = 1.89, *p* = 0.15, *η*^2^ = 0.002; empathy, F_3,1876_ = 1.11, *p* = 0.34, *η*^2^ = 0.002; familiarity, F_3,1854_ = 0.36, *p* = 0.78, *η*^2^ = 0.001; favorability, F_3,1854_ = 2.5, *p* = 0.06, *η*^2^ = 0.004) and no significant interactions. For the four images, subjective scores (emotional arousal, familiarity, empathy, and favorability for each) were combined by averaging. This process was used for simplifying the data to reduce the number of variables, and to confirm the absence of differences between the two female images and the two male images, and between the female and male images.

Statistical reports of interaction follow-up analysis

We conducted a two way ANOVA to test for main and interaction effects, and followed with Bonferroni (BF)-corrected comparison using estimated marginal means (EMMEANS) in SPSS. Additionally, uncorrected p value and false discovery rate (FDR)-corrected p value were presented.

Supplementary Table 1. Statistical analysis results for **emotional arousal** in multiple comparisons between emotion types.

Supplementary Table 2. Statistical analysis results for **emotional arousal** in multiple comparisons between conditions.

Supplementary Table 3. F values represent simple effects of emotion type (six levels) within condition (three levels) on **emotional arousal**, based on pairwise comparisons of EMMEANS.

Supplementary Table 4.

EMMEANS of **emotional arousal** for the interaction between condition (three levels) and emotion type (six levels). Pairwise comparisons of emotion types were conducted within each condition. BF adjusted, uncorrected and FDR corrected p values are reported.

Supplementary Table 5. F values represent simple effects of condition (three levels) tested separately within each of the emotion type (six levels) on **emotional arousal**, based on pairwise comparisons of EMMEANS.

Supplementary Table 6.

EMMEANS of **emotional arousal** for the interaction between condition (three levels) and emotion type (six levels). Pairwise comparisons of conditions were conducted within each emotion type. BF adjusted, uncorrected and FDR corrected p values are reported.

Supplementary Table 7.

Statistical analysis results for **familiarity** in multiple comparisons between emotion types.

Supplementary Table 8.

Statistical analysis results for **familiarity** in multiple comparisons between conditions.

Supplementary Table 9.

F values represent simple effects of emotion type (six levels) within condition (three levels) on **familiarity**, based on pairwise comparisons of EMMEANS.

Supplementary Table 10.

EMMEANS of **familiarity** for the interaction between condition (three levels) and emotion type (six levels). Pairwise comparisons of emotion types were conducted within each condition. BF adjusted, uncorrected and FDR corrected p values are reported.

Supplementary Table 11.

F values represent simple effects of condition (three levels) tested separately within each of the emotion type (six levels) on **familiarity**, based on pairwise comparisons of EMMEANS.

Supplementary Table 12.

EMMEANS of **familiarity** for the interaction between condition (three levels) and emotion type (six levels). Pairwise comparisons of conditions were conducted within each emotion type. BF adjusted, uncorrected and FDR corrected p values are reported.

Supplementary Table 13. Statistical analysis results for **empathy** in multiple comparisons between emotion types.

Supplementary Table 14. Statistical analysis results for **empathy** in multiple comparisons between conditions.

Supplementary Table 15. F values represent simple effects of emotion type (six levels) within condition (three levels) on **empathy**, based on pairwise comparisons of EMMEANS.

Supplementary Table 16.

EMMEANS of **empathy** for the interaction between condition (three levels) and emotion type (six levels). Pairwise comparisons of emotion types were conducted within each condition. BF adjusted, uncorrected and FDR corrected p values are reported.

Supplementary Table 17.

F values represent simple effects of condition (three levels) tested separately within each of the emotion type (six levels) on **empathy**, based on pairwise comparisons of EMMEANS.

Supplementary Table 18.

EMMEANS of **empathy** for the interaction between condition (three levels) and emotion type (six levels). Pairwise comparisons of conditions were conducted within each emotion type. BF adjusted, uncorrected and FDR corrected p values are reported.

Supplementary Table 19. Statistical analysis results for the effect of **favorability** in multiple comparisons between emotion types.

Supplementary Table 20. Statistical analysis results for the effect of **favorability** in multiple comparisons between conditions.

Supplementary Table 21. F values represent simple effects of emotion type (six levels) within condition (three levels) on **favorability**, based on pairwise comparisons of EMMEANS.

Supplementary Table 22.

EMMEANS of **favorability** for the interaction between condition (three levels) and emotion type (six levels). Pairwise comparisons of emotion types were conducted within each condition. BF adjusted, uncorrected and FDR corrected p values are reported.

Supplementary Table 23.

F values represent simple effects of condition (three levels) tested separately within each of the emotion type (six levels) on **favorability**, based on pairwise comparisons of EMMEANS.

Supplementary Table 24.

EMMEANS of **favorability** for the interaction between condition (three levels) and emotion type (six levels). Pairwise comparisons of conditions were conducted within each emotion type. BF adjusted, uncorrected and FDR corrected p values are reported.

Supplementary Table 25.

Multiple comparisons of fR. Statistical analysis results for the effects of emotion type on fR.

Supplementary Table 26.

Multiple comparisons of fR. Statistical analysis results for the effects of condition on fR.

Supplementary Table 27. Multiple comparisons of fR.

Statistical analysis results for the effect of condition on fR compared between emotion types.

Supplementary Table 28. Multiple comparisons of fR.

Statistical analysis results for the effect of emotion type on fR compared between conditions.

Supplementary Table 29. Multiple comparisons of HR.

Statistical analysis results for the effects of emotion on HR.

Supplementary Table 30.

Multiple comparisons of HR.

Statistical analysis results for the effects of emotion type (a) and condition (b) on HR.

Supplementary Table 31. Multiple comparisons of HR. Statistical analysis results for the effect of condition on HR compared between emotion types.

Supplementary Table 32. Multiple comparisons of HR.

Statistical analysis results for the effect of emotion type on HR compared between conditions.

Supplementary Table 33.

Supplementary Fig. 1. Exploratory analysis taking sex differences into account

In an exploratory analysis, sex-related factors were included as independent variables in a three-way ANOVA. The three-way ANOVA was performed with subjective score as the dependent variable, and type of emotion, condition, and sex as independent variables.

The overall results were the same as those of the main analysis, indicating that empathy scores for joy stimuli were higher in the synchro condition compared with those in the static and asynchro conditions, for both female and male study participants. Empathy scores for joy stimuli were higher in the synchro condition compared with those in the static and asynchro conditions, and compared with those in the synchro conditions for all other emotion types. The degree of emotional arousal, empathy, familiarity, and favorability for joy stimuli were not significantly different between female and male study participants. Differences were observed in the main effects of sex on empathy and favorability. Empathy for anger and sadness stimuli was higher in all conditions in male study participants (all *p*-values < 0.05) compared with that in female study participants, and favorability scores for anger stimuli were higher for all conditions in male study participants compared with those in female study participants (*p* < 0.05).

**Supplementary Methods**

Milli-wave radar

We used a frequency modulated continuous wave (FMCW) radar system (RFR79ITR34-30U, PTM Corp, Yokohama) operating from 79 GHz. The system used a beam width of 15° vertically and 76° horizontally, with a fan-shaped radiation beam (IWR 1843/IWR6843, Texas Instrument) to measure study participant’s respiration (Supplementary Fig. 2).

The radar system consists of milli-wave radar blocks: one transmitter and one receiver, and a programmed MCU with a hardware accelerator for radar signal processing, including distance fast Fourier transform (FFT) spectrum, velocity FFT, azimuth (direction) information, and elevation information. The product specifications of the radar system used in this study are shown in Supplementary Table 33 and Fig. 2.

In this system, an electromagnetic wave is sent from the transmitter into an environment containing various objects. The echo of the wave is captured by the receiver. A milli-wave system operating at 79–81 Hz (corresponding to a wavelength of approximately 4 mm) has the ability to detect movements as small as 0.1 mm. For a typical adult, the chest moves approximately 1–12 mm and 0.01–0.5 mm during breathing and heartbeat, respectively ^Suppl.Ref.1^,. The chest wall and abdomen move forward (during inhalation) and backward (during exhalation) during respiratory activity. The use of FMCW radar for detecting human vital signs has been well studied in the field of biomedical engineering ^Suppl.Ref.1,2^.

A brief description of the algorithm is as follows. In FMCW, the frequency of the signal increases or decreases linearly with time, and is called a chirp. A chirp is a signal that sweeps across a range of frequencies over a specific duration. This transmitted signal hits a target, and the signal is reflected back to the receiver. The transmitted signal continuously changes frequency, and the frequency of the received signal differs from the current transmitted signal because of the time delay. This frequency difference is called the beat frequency, and is proportional to the distance of the target. The radar first determines the range to the study participant using the beat frequency from the FMCW signal, and this range measurement isolates the study participant from other objects in the environment. Objects such as walls or furniture are identified as static objects, and are distinguished from the study participant’s body. The radar can detect small movements of the chest wall caused by breathing. The small movements cause minute changes in the distance between the radar and the study participant and alter the phase of the reflected signal. Spectral analysis using FFT was applied to evaluate the depth (distance) between the milli-wave radar and the participants. Additionally, a filter was developed to improve the signal-to-noise ratio in respiratory movement estimation, decomposing the signal into its frequency components. By analyzing the frequency content of the signal, the radar can identify the characteristic frequencies of breathing (0.5 Hz to 1.5 Hz). Detailed mathematical formula have been reported elsewhere ^Suppl.Ref.1,2^.

Supplemental references

# 1 Wang, Y., Wang, W., Zhou, M., Ren A., Tian, Z. Remote monitoring of human vital signs based on 77-GHz mm-Wave FMCW Radar. Sensors (Basel), 20,10, 2999 (2020) **doi:**[**10.3390/s20102999**](https://doi.org/10.3390%2Fs20102999)**.**

# **2 Kebe, M., Gadhafi, R., Mohammad, B., Sanduleanu, M., Saleh, H., A1-Qutayri, M. Human vital signs detection method and potential using radars; A review. Sensors, 20, 5, 1454 (2020) doi:**[**10.3390/s20051454**](https://doi.org/10.3390%2Fs20051454)

Supplementary Fig. 2

Confirmation of respiratory movement accuracy measured with milli-wave radar

To confirm the accuracy of measurement of respiration with milli-wave radar, we investigated the similarity between respiratory movement measured with milli-wave-radar and that measured with a respiratory band device (TN 1132/ST, AD Instrument, Aichi, Japan). Simultaneous recording of these two measurements was performed using PowerLab (ML846; ADInstruments, Aichi, Japan) and presented on a chart (Supplementary Fig. 3). Cross correlation analysis was performed for two waves, indicating a high level of similarity between them (r = 0.97).

Supplementary Fig. 3

**Video description**

Supplementary Video S1. Representative video of the static-condition. Facial expression images originally displayed on the monitor have been replaced with non-identifiable schematic illustrations for display purposes.

Supplementary Video S2. Representative video of the asynchro-condition. Facial expression images originally displayed on the monitor have been replaced with non-identifiable schematic illustrations for display purposes.

Supplementary Video S3. Representative video of the synchro-condition. Facial expression images originally displayed on the monitor have been replaced with non-identifiable schematic illustrations for display purposes.
